# Caesarean Delivery in South Italy: Women without Choice. A Cross Sectional Survey

**DOI:** 10.1371/journal.pone.0043906

**Published:** 2012-09-17

**Authors:** Pamela Barbadoro, Carlos Chiatti, Marcello Mario D’Errico, Francesco Di Stanislao, Emilia Prospero

**Affiliations:** Department of Biomedical Sciences, Università Politecnica delle Marche, Ancona, Italy; Tehran University of Medical Sciences, Iran (Republic of Islamic)

## Abstract

**Background:**

In spite of the World Health Organization’s recommendations to maintain caesarean delivery (CD) between 5% and 15% of total births, the rates of CD continue to rise in countries with routine access to medical services. As in Italy CD rate reached 38% in 2008, the highest at EU level, we evaluated socioeconomic and clinical correlates of “elective” and “non programmed” CD in the Country. We performed a stratified analysis in order to verify whether the effect of such correlates differed among women with an “*a priori”* preference for natural and caesarean delivery respectively.

**Methods and Findings:**

We analyzed cross-sectional data from the Italian National Statistics Institute (ISTAT) survey on health condition. Socio-demographic variables, information on maternal care services use and health conditions during pregnancy, as well as maternal preferences on delivery, were available for a representative sample of 2,474 primiparous women. After an initial bivariate analysis, we used logistic regressions to evaluate factors associated to the study outcomes. Overall CD accounted for 35.5% of the total births in our sample (CI 33.6–37.4%); moreover, 30.7% (CI 28.6–32.6%) of women preferring natural delivery actually delivered with a CD. Elective CD rate is higher among women over 35 years (22.9%, CI 18.8–27.4%), and those living in the South (26.2%, CI 23.0–29.6%). The multivariate analysis showed that, even adjusting for several confounders, women in the South, receiving care in the private sector had higher chances of CD, also in case of preference for natural delivery.

**Conclusion:**

Policy interventions are required to reduce the rate of undesired CD, e.g. increasing women knowledge regarding delivery in order to favour aware choices. An effective strategy to reduce CD rate should address the Southern Regions, as women here appear to have a very limited control over the delivery, in spite of a widespread preference for natural delivery.

## Introduction

The World Health Organization recommends to maintain caesarean delivery (CD) between 5% and 15% of total births: the lower limit represents the expected rate of interventions to prevent maternal mortality and/or severe morbidity, the upper is the limit above which no improvements in maternal and neonatal health outcomes are observed [1].

In spite of WHO recommendations, the rates of CD continue to rise in countries with routine access to medical services, reaching about the 20–25% of total births [2]; [3]; [4]. In Italy, given the increase of CD rate from 35.3% in 2002 up to 38.0% in 2008, the highest at the EU level [5], the Ministry of Health has recently updated specific National Guidelines for an aware choice of CD [6]. The medical establishment has been recently discussing about caesarean section on maternal request [7]; [8]; [9]. However, little is known about what influences the request and whether a preference expressed in early pregnancy may be changed in the light of additional information; moreover, we do not know how such requests are managed by obstetric teams in different healthcare settings [8].

In this study, we investigated the socioeconomic and healthcare variables associated to CD in Italy. Moreover, variables concerning women preferences about CD *a priori* were analysed in relation to the actual mode of delivery.

## Methods

### Study Design

A cross-sectional study was conducted using data from the last available edition of the multipurpose survey “Health and health care use”. This survey is performed by the Italian National Institute of Statistics (ISTAT), and aims at investigating on a variety of aspects concerning the health of the population, e.g., the prevalence of chronic diseases, the lifestyles and the patterns of health care use. The health survey is performed to monitor health care needs, and use of healthcare services, mainly for health policy matters. In order to ensure the representativeness of the sample, a stratified multi-stage probability design was used to select a sample using municipal lists of households. In the first stage, municipalities were the primary sampling units. Municipalities were selected from 68 strata defined on a regional basis and based on population size; a minimum of four municipalities were selected for each stratum. The municipalities were further stratified into: 1) larger and metropolitan municipalities, and 2) smaller municipalities. The minimum population size used to include a municipality among the socalled “larger municipalities” (r^λ^ ) varied within each stratum; in particular, this threshold (r^λ^ ) was calculated according to the following formula: r^λ^ = r^m^r^δ^/r^f^, where r^m^ represents the minimum number of households to be interviewed in each municipality of the sample, r^δ^ indicates the mean number of household members in the relative strata, and r^f^ stands for the sampling fraction [9]; for instance, the range of population size included in the larger municipalities was between 4,000 and 47,000 inhabitants. All larger municipalities were included in the sample; whereas for smaller municipalities, a sample municipality within each stratum was extracted with a probability proportional to the target population size, according to the systematic selection procedure by Madow [10]. From a total of 8092, 1476 municipalities were selected from the larger (N = 281) and smaller municipalities (N = 1,195).

The second stage of the sample design involved clustering households from municipality lists. The sampling unit was a household of persons living together, with legal, affective, or family relationships, without regard to number of persons in the household. Within each municipality, a minimum of 30 households were randomly selected to be included in the study. Sampling continued without replacement until the required sample size was achieved. Exclusion criteria were: those household members who had died, residence outside of Italy or in a residential care structure, nonexistent address, and inability to localize or access the address. The mean eligibility rate for the 2004–2005 survey was 96.45% [11]. The overall non-response rate was 17% and was mostly attributable to refusals (approximately 9% of the total sample), whereas 5% were list errors, and other reasons for non-response accounted for 3% of the sampled households [12]. More detailed information on quality on the execution of ISTAT Multipurpose survey, together with process details can be found at the ISTAT website [13].

### Study Sample

The last edition, performed between December 2004 and September 2005, gathered data on 50,474 families and 128,040 individuals, representative in terms of age, gender and geographical distribution of the Italian population. These survey included information on maternal and neonatal health in a sample of 5 812 women who had had a pregnancy in the five years before the interview [10]. Our analysis focused on a subsample of 2 474 primiparous women who were asked about the type of delivery, i.e. “natural”, “elective” and “non programmed” caesarean. The study of women preferences for their type of delivery, *a priori*, has been studied by analysing the answer to the question: “If you had the possibility, what type of delivery would you choose?”, with the following codified answers: “natural delivery”, or “caesarean delivery”. All data used in this analysis are released by ISTAT in anonymous form, therefore the approval of the competent Ethics Committee was not required for this specific study. In order to assure anonymity, no tabular data with total sum of 10 or less in small geographic areas will be reported.

### Study Variables

For all sampled subjects socio-demographic characteristics, information on maternal care service utilisation and on selected health conditions during pregnancy were available.

Among the socio-demographic characteristics we considered age and geographical area of residence classified as North-West; North-East; Central; South; Islands. Socioeconomic status was assessed by using educational level, classified as low (none or comprehensive school), medium-low (intermediate school), medium-high (high school degree), high (university degree). In addition, a neoweberian classification of social classes previously validated in Italy [14], which utilises occupation as a proxy of the social position, was used; this procedure identifies four classes: lower (skilled and unskilled working class), middle-lower (self-employed without employees); middle-upper (white collars and small employers); and upper class. Individuals with no working experience, e.g. students, housewives, and unemployed, were grouped in a fifth residual category. In a following step, household social class was defined selecting the highest individual social class within the family [15].

The following information was retrieved: a) place of delivery (“Public” hospital = 0 Vs. “Private” = 1); b) physician who attended the pregnancy (“Public” = 0 Vs. “Private” = 1 Obstetrician); c) antenatal diagnostic services used, and the characteristics of the providers (recoded as “no diagnostics” = 0, “public diagnostic facility” = 1, and “private diagnostic facility” = 2); d) antenatal class attendance (Not attended = 0; Attended = 1). We considered those pregnancy-related conditions that could be associated to risk of CD, assuming that risk assessment in pregnancy is not a once-only measure, but a procedure continuing throughout pregnancy and labour [16]. The following selected conditions were available from the national survey, and considered as possibly related to higher risk of CD: Diabetes during pregnancy (No = 0; Yes = 1); Hypertension during pregnancy (No = 0; Yes = 1); Risk of Pre-term delivery (delivery before 37 weeks of gestation, No = 0; Yes = 1); Risk of Abortion (No = 0; Yes = 1); Hospitalisation during pregnancy (No = 0; Yes = 1). Our analysis included also newborn’s birthweight as this variable is strongly dependent on fetal growth and pregnancy duration.

### Statistical Methods

Initial bivariate analysis was performed to analyse the changes in caesarean delivery rates according to all investigated variables. In a second step, multinomial logistic regression analysis were used to evaluate factors independently associated to a)“elective”, and b) “non programmed” CD. Two logistic models were also used to identify variables associated to c) CD in women who expressed *a priori* preference for natural delivery, and d) CD in women who expressed *a priori* preference for this type of delivery. Socio-demographic characteristics, information on maternal care service utilisation, and on selected health conditions during pregnancy were used to assess the models. In order to take into account the role of gestational age at delivery on birth-weight, the delivery of a baby before 37 weeks of gestation has been defined as preterm delivery. Since the study and sampling design, and the federalist reforms which have given different regions a high degree of autonomy in organising local health care systems, multilevel regression models were used in order to consider the connection of the outcome variable at area-level. Covariates were included into the models with a stepwise procedure. Explanatory variables that were associated with the outcome at a significance of 0.20 or less at univariate analysis, were included as independent variables to adjust for the indirect effects of other variables. Association between the characteristics and study outcomes was expressed as Odds Ratios (ORs) and 95% confidence intervals (CIs). Standard post-estimation tests were used to assess the models validity, i.e. F-statistics and ROC curves observation. Level of significance was set at 0.05. Analyses were performed using STATA, version 9, software.

**Table 1:** Elective and non-programmed Caesarean Section rates according to main sociodemographic characteristics (n = 2 474).

			Primiparous women				Women preferring natural delivery			Women preferring Cesarean		
		(n)	% natural delivery	% Elective CD	% non-progr. CD	p	(n)	% CD	p	(n)	% CD	p
	Total sample	2474	64.5	16.4	19.1		2162	30.7		312	68.6	
Maternal age	14–19	78	69.3	11.5	19.2	<0.001	67	25.4	<0.001	11	63.6	0.209
	20–34	2011	66.2	15.4	18.4		1773	29.4		238	66.4	
	35 and more	385	54.5	22.9	22.6		322	39.1		63	77.8	
Educational level A	Low	100	61	18	21	0.534	82	34.2	0.78	18	61.1	0.573
	Medium – low	765	64.9	17.1	18		663	29.6		102	71.6	
	Medium – high	1192	64.7	15.1	20.2		1047	30.6		145	69.7	
	High	417	64.2	18.5	17.3		370	32.4		47	61.7	
Family social class	Lower	693	68.3	14.4	17.3	0.223	609	26.8	0.083	84	67.9	0.958
	Middle – lower	270	61.8	18.2	20		239	34.3		31	67.7	
	Middle – upper	860	65	15.5	19.5		756	30.6		104	67.3	
	Upper	538	61.1	18.6	20.3		462	33.8		76	69.7	
Area of residence	Northern – west	506	69.5	12.1	18.4	<0.001	449	25.8	<0.001	57	66.7	0.026
	Northern – east	528	70.8	10.6	18.6		479	26.3		49	57.1	
	Central	469	69.5	11.9	18.6		422	27.3		47	59.6	
	Southern	710	53.1	26.2	20.7		591	40.4		119	79	
	Island	261	64	18	18		221	30.8		40	65	
Professional attending the pregnancy	Public Obstetrician	382	71.7	12.3	16	0.003	1791	25.4	0.006	270	57.1	0.301
	Private Obstetrician	2031	62.8	17.4	19.8		347	35.1		35	70	
Place of delivery	Public hospital	2418	65	15.8	19.2	<0.001	2118	30.4	0.032	300	68.3	0.626
	Private outside NHS	56	48.2	41.1	10.7		44	45.5		12	75	
Antenatal diagnostic	Not done	279	67.7	16.9	15.4	0.009	236	26	0.072	44	65.9	0.227
	Public	1600	65.7	14.8	19.5		1419	30.3		181	65.8	
	Private	595	59.8	20.5	19.7		508	34.1		87	75.9	
Antenatal class	No	1367	59.2	21.4	19.4	<0.001	1150	34.6	<0.001	217	73.7	0.003
	Yes	1107	71.1	10.2	18.7		1012	26.3		95	56.8	
Diabetes	No	2422	64.6	16.3	19.1	0.629	2115	30.6	0.412	307	68.4	0.579
	Yes	52	59.6	21.2	19.2		47	36.2		5	80	
Hypertension	No	2359	65.4	16.2	18.4	<0.001	2066	29.9	<0.001	293	67.9	0.316
	Yes	115	46.1	21.7	32.2		96	49		19	79	
Pre-term delivery risk	No	2211	65.7	16	18.3	0.001	1939	29.4	<0.001	272	69.1	0.6
	Yes	263	54.3	19.8	25.9		223	42.2		40	65	
Abortion risk	No	2163	65.5	16	18.5	0.023	1901	29.6	0.002	262	70.2	0.153
	Yes	311	57.6	19.3	23.1		261	39.1		50	60	
												
Hospitalisation	No	1366	67.7	16.3	16	<0.001	1209	27.4	<0.001	157	69.4	0.749
	Yes	1108	60.5	16.6	22.9		953	34.9		155	67.7	
Gestational age at birth	<27 weeks	9	33.3	22.2	44.4		9	66.7		–	–	
	27–31 weeks	42	23.8	19.1	57.1		33	72.3		9	88.9	
	32–36 weeks	163	47.2	30.1	22.7		129	43.4		34	88.2	
	>37 weeks	2260	66.6	15.4	18.0	<0.001	1991	29.0	<0.001	269	65.4	0.011
Birthweight	>2,500 grams	2292	66.2	15.9	17.9	<0.001	2020	29.3	<0.001	272	67.3	0.193
	<2500 grams	177	43.5	23.7	32.8		137	50.4		40	77.5	

A Educational level was categorized as follows: Low (ISCED = 0. 1). Medium-low (ISCED = 2). Medium-upper (ISCED = 3. 4). Upper (ISCED = 5. 6);

## Results

Although only 12.6% of participants (N = 312) declared their preference for CD, the overall CD accounted for 35.5% (CI, 33.6–37.4%, N = 878) of the total births in our sample (Table 1).

**Table 2:** Results of multinomial logistic regression models for estimated of factors associated with elective and non-programmed Caesarean Section in a representative sample of Italian mothers (N = 2 469).

	*Elective Cesarean Section*				*Non-programmed Cesarean Section*			
	*(406 out of 2474)*				*(472 out of 2474)*			
	*OR*	*(95% CI)*		*p*	*OR*	*(95% CI)*		*p*
Maternal age (one year increase)	**1.10**	**1.07**	**1.12**	**0.00**	**1.03**	**1.01**	**1.06**	**0.01**
Educational level A								
- High	1				1			
- Low	1.03	0.55	1.92	0.94	0.94	0.53	1.65	0.82
- Medium-low	1.00	0.48	1.68	0.74	0.99	0.56	1.76	0.99
- Medium-high	0.88	0.44	1.74	0.71	0.74	0.39	1.39	0.35
Family social class								
- Upper	1				1			
- Middle – upper	0.87	0.56	1.37	0.56	0.89	0.59	1.35	0.59
- Middle – lower	0.82	0.60	1.14	0.25	0.88	0.58	1.32	0.54
- Lower	**0.67**	**0.45**	**0.98**	**0.04**	0.72	0.51	1.02	0.07
Area of residence								
- Northern-west	1				1			
- Northern-east	0.92	0.61	1.39	0.68	1.00	0.71	1.39	0.99
- Central	0.94	0.62	1.43	0.77	1.07	0.76	1.51	0.72
- Southern	**2.78**	**1.94**	**4.00**	**0.00**	**1.56**	**1.12**	**2.17**	**0.01**
- Island	**1.64**	**1.03**	**2.62**	**0.04**	1.28	0.84	1.95	0.25
Professional who attended the pregnancy								
- Public Obstetrician	1				1			
- Private Obstetrician	1.28	0.89	1.83	0.18	1.20	0.88	1.64	0.24
Place of delivery (private Vs. public hospital)	**2.05**	**1.07**	**3.93**	**0.03**	0.66	0.26	1.65	0.38
Antenatal diagnostic								
- Not done	1				1			
- In a Public facility	0.86	0.58	1.27	0.46	1.21	0.83	1.76	0.33
- In a Private facility	1.17	0.76	1.80	0.48	1.27	0.84	1.93	0.26
Antenatal class attendance	**0.64**	**0.54**	**0.76**	**0.00**	**0.86**	**0.76**	**0.99**	**0.03**
Health-related problems during pregnancy								
- Diabetes	0.88	0.40	1.96	0.76	0.66	0.31	1.42	0.29
- Hypertension	**2.23**	**1.25**	**3.98**	**0.01**	**1.90**	**1.17**	**3.01**	**0.01**
- Pre-term delivery risk	1.16	0.76	1.77	0.50	1.11	0.76	1.62	0.57
- Pre-term abortion risk	1.26	0.85	1.88	0.25	1.01	0.71	1.43	0.95
Hospitalisation during pregnancy	0.93	0.69	1.25	0.63	**1.48**	**1.15**	**1.90**	**0.02**
Birthweight B								
≥2,500 grams	**1**				**1**			
<2500 grams	1.29	0.77	2.18	0.33	**2.08**	**1.33**	**3.26**	**0.00**
Pre-term birth	**2.69**	**1.75**	**4.13**	**0.00**	**1.67**	**1.01**	**2.54**	**0.02**

OR = Odds Ratio; CI = Confidence Interval.

A Educational level was categorized as follows: Low (ISCED = 0. 1). Medium-low (ISCED = 2). Medium-upper (ISCED = 3. 4). Upper (ISCED = 5. 6);

B Children weighting less than 2 500 grams were classified as low birthweight.

Crude rates of elective and non programmed CD were 16.4% (CI, 15.0–17.9%, N = 406) and 19.1% (CI, 17.6–20.7%, N = 472) respectively. A total of 661 women out of 2162 (30.7%, CI 28.6–32.6%) preferring natural delivery actually delivered with a Caesarean Section. Elective CD rate is higher among the women aged over 35 years (22.9%, CI, 18.8–27.4%, N = 88), and those living in the Southern regions (26.2%, CI, 23.0–29.6%, N = 186). The highest crude rate of elective CD is among the women who delivered in a private hospital (41.1%, CI 28.1–55.0%, N = 23).

**Table 3:** Results of multinomial logistic regression models for estimate of factors associated with Caesarean Section in subgroups of women preferring Natural and Cesarean delivery respectively.

	*Preference for natural. but cesarean delivery*						*Preference for cesarean and cesarean delivery*					
	*(664 out of 2162)*						*(214 out of 312)*					
	*OR*		*(95% CI)*		*p*		*OR*		*(95% CI)*		*p*	
Maternal age (one year increase)	**1.06**	**1.03**		**1.09**		**0.00**	**1.08**	**1.04**		**1.12**		**0.00**
Educational level A												
- Low	1						1					
- Medium-low	0.78	0.41		1.47		0.44	1.08	0.52		2.26		0.83
- Medium-high	0.73	0.39		1.39		0.34	0.91	0.43		1.90		0.80
- High	0.85	0.43		1.71		0.65	0.59	0.25		1.35		0.21
Family social class												
- Upper	1						1					
- Middle – upper	1.10	0.68		1.77		0.71	0.58	0.33		1.03		0.06
- Middle – lower	0.95	0.67		1.35		0.76	0.75	0.50		1.13		0.17
- Lower	0.81	0.53		1.23		0.32	0.67	0.42		1.06		0.09
Area of residence												
- Northern-west	1						1					
- Northern-east	0.98	0.62		1.55		0.93	0.74	0.44		1.26		0.27
- Central	1.03	0.65		1.64		0.89	0.76	0.45		1.30		0.32
- Southern	**2.29**	**1.54**		**3.40**		**0.00**	**1.61**	**1.04**		**2.50**		**0.03**
- Island	1.65	0.99		2.73		0.05	1.31	0.75		2.31		0.34
Professional who attended the pregnancy												
- Public Obstetrician	1						1					
- Private Obstetrician	1.00	0.68		1.47		0.99	1.55	0.95		2.54		0.08
Place of delivery (private Vs. public hospital)	1.88	0.96		3.68		0.06	1.68	0.77		3.64		0.19
Antenatal diagnostic												
- Not done	1						1					
- In a Public facility	0.97	0.63		1.51		0.91	**0.62**	**0.39**		**0.98**		**0.04**
- In a Private facility	1.35	0.84		2.17		0.22	0.89	0.54		1.48		0.67
Antenatal class attendance	**0.75**	**0.63**		**0.89**		**0.00**	**0.62**	**0.49**		**0.77**		**0.00**
Health-related problems during pregnancy												
- Diabetes	0.99	0.42		2.34		0.98	0.58	0.19		1.75		0.33
- Hypertension	**1.77**	**1.00**		**3.15**		**0.05**	1.36	0.71		2.63		0.36
- Pre-term delivery risk	1.24	0.79		1.94		0.35	0.80	0.48		1.36		0.41
- Pre-term abortion risk	1.23	0.81		1.88		0.33	0.89	0.55		1.45		0.64
Hospitalisation during pregnancy	0.81	0.59		1.11		0.38	1.25	0.87		2.67		0.14
Birthweight (normal Vs low) B												
≥2,500 grams	**1**						1					
<2,500 grams	0.77	0.43		1.37		0.28	1.52	0.87		2.67		0.14
Pre-term birth	**1.72**	**1.08**		**2.73**		**0.02**	**2.22**	**1.35**		**3.65**		**0.00**

OR = Odds Ratio; CI = Confidence Interval.

A Educational level was categorized as follows: Low (ISCED = 0. 1). Medium-low (ISCED = 2). Medium-upper (ISCED = 3. 4). Upper (ISCED = 5. 6);

B Children weighting less than 2 500 grams were classified as low birth weight.

At multivariate analysis (Table 2), factors independently associated with elective CD were higher maternal age (one year increase, OR = 1.10; CI 1.07–1.12), living in the South (OR = 2.78; CI 1.94–4.00), or in the Islands (OR = 1.64, CI 1.03–2.62), delivering in a private hospital (OR = 2.05; CI 1.07–3.93). Women belonging to the lower social classes had lower odds of elective CD (OR = 0.67; CI 0.45–0.98), such as those having attended antenatal classes (OR = 0.64; CI 0.54–0.76). Preterm birth was a strong determinant of both elective (OR = 2.69; CI 1.75–4.13), and non-programmed CD (OR = 1.67 CI 1.01–2.54).

Higher maternal age (OR = 1.03; CI 1.01–1.06), and living in the South (OR = 1.56; CI 1.12–2.17) represented risk factors for non-programmed CD, as well as unscheduled hospitalisation during pregnancy (OR = 1.48; CI 1.15–1.90). Hypertension was associated to a higher likelihood of both elective (OR = 2.23; CI 1.25–3.98) and non-programmed CD (OR = 1.90;CI 1.17–3.01). Moreover, non-programmed CD was associated to pre-term birth (OR 1.67, CI 1.01–2.54), and low birth weight (OR 2.08, CI 1.33–3.26). On the other hand, antenatal class attendance was associated to a reduced risk of non-programmed CD (OR 0.86, CI 0.76–0.99).

As for the factors associated to delivering by CD among women with a *“a priori”* preference for natural delivery (Table 3) were increasing maternal age (OR 1.06, 95%CI 1.03–1.09), living in Southern areas of the Country (OR 2.29, CI 1.54–3.40), hypertension during pregnancy (OR 1.77, CI 1.00–3.15), and pre-term delivery (OR 1.72, CI 1.08–2.73). The participation in antenatal classes was associated to a reduced risk of CD (OR 0.75, CI 0.63–0.89). As for women preferring CD *“a priori*” (Table 3), living in the Southern area of the country (OR 1.61, CI 1.04–2.50), and preterm birth were associated to an increased risk of CD (OR 2.22, CI 1.35–3.65). Conversely, receiving prenatal care from a public facility (OR 0.62, CI 0.39–0.98), together to antenatal class attendance were related to a reduced frequency of CD (OR 0.62, CI 0.49–0.77).

## Discussion

In our study, 12.6% of women declared to prefer *a priori* a CD to a natural delivery. This proportion is in line with the results recently reported by a systematic review of published studies which estimated a 10.1% (95% CI 7.5–13.1) of women without a previous caesarean section preferring a CD [17]. However, a considerable proportion (such as 30.7%) of women preferring natural delivery seems to have been re-directed or to have changed their mind, and delivered using Caesarean Section. In our study, an incremental risk of caesarean for the women living in the South, and in the Islands, could not be explained by any variable included in the model. This suggested that if maternal or professionals’ preferences for CD delivery exist, they “cluster” locally, confirming what Mancuso and colleagues [18] already asserted, i.e. that in Italy there are “loco-regional factors” related to socio-cultural and healthcare background influencing maternal choice. These differences in the patterns of health care use have to be considered carefully as the federalist reforms occurring in Italy are devolving increasing responsibilities to the regional authorities, who have now to cover possible deficits of the health care system with their own resources (for instance by increasing the regional taxation).

Above considerations seem even more important when considering that the ORs of delivering with CD in women with an “a priori” preference for natural delivery, where reduced after having received antenatal diagnostics in a public facility, thus stressing the potential role of health care facility/professionals in determining CD in women with different expectations. The role of women, and healthcare professionals in deciding about the mode of delivery, may be highlighted by the fact that birth weight under 2,500 grams was associated only to non-programmed CD, while the choice of women having CD when preferring natural was not associated to the newborn weight.

A large number of studies so far have investigated on the role of maternal preferences in determining CD and on the ways through which preferences interact with health professionals views. Recently, the effect of maternal requests on the increase of CD has been critically reviewed by Gamble and colleagues [19], who suggested that few women request a CD in the absence of current or previous obstetric complication. These findings should be an input for future researches inspecting the role of health conditions of the mother and the foetus, the knowledge, expectations, and beliefs of the future parents in order to deeply understand this important event.

The analysis of CD on a nationwide, representative basis has provided the opportunity to highlight the influence of different healthcare organization on a regional basis (i.e. with region of the South of Italy having a more frequent offer of private facilities with respect to those belonging to the Northern areas of the Country). Nevertheless, this study has some limitations that must be considered. First, the cross-sectional design of the survey enabled us to determine only simple epidemiologic associations between variables rather than cause-effect relationships.

Second, the study is an observational one, and even if we included in models important covariates, residual confounding may have influence results. In this regard, it must be remarked that during statistical analysis, we explored the effect of several variables not included in final models, such as household wealth, housing characteristics. However, no meaningful changes occurred in our results. Furthermore, Patel and colleagues [20] realised a similar study using data from a large cohort of pregnant women in the UK, obtaining results largely consistent with ours.

Despite these caveats, our analysis shows that, even after adjusting for several confounders, women living in the South, undergoing prenatal care in private facilities and delivering in a private hospital have higher chances of CD, also if they would have preferred natural delivery. If these findings will be confirmed by further analysis, policy interventions should be considered in order to reduce the rate of unwanted CD, and give much more information and support to make CD an aware decision of women.
